# Nematode community structure along elevation gradient in high altitude vegetation cover of Gangotri National Park (Uttarakhand), India

**DOI:** 10.1038/s41598-022-05472-9

**Published:** 2022-01-26

**Authors:** Priyanka Kashyap, Shahid Afzal, Anjum Nasreen Rizvi, Wasim Ahmad, V. P. Uniyal, Dhriti Banerjee

**Affiliations:** 1 Wildlife Institute of India, Chandrabani, Dehradun, Uttarakhand 248001 India; 2 Zoological Survey of India, New Alipore, Kolkata, West Bengal 700053 India; 3 Nematode Biodiversity Research Laboratory, Department of Zoology, Aligarh Muslim University, Aligarh, 202002 India

**Keywords:** Ecology, Zoology

## Abstract

The study was undertaken to understand the dynamics of soil nematode communities association along elevation gradient and associated variables in high-altitude regions of Western Himalaya. The diversity and distribution pattern of soil nematodes were investigated at four elevation ranges (Elv1, Elv2, Elv3, and Elv4 each of 500 m class) along altitudinal gradient (3000–5000 m). The nematode community comprised 58 genera of which 37 genera showed sensitivity towards altitudinal variation and the community structure also varied among elevation classes. It was found that elevation significantly affect the nematodes trophic group, diversity composition, and the nematode indices. Nematode generic richness and evenness index indicated a relatively low biodiversity of soil nematodes is supported at higher altitudes. Moreover, maturity indices reflected lower sustenance of k-strategic nematodes at higher elevations. Structure index depicted fewer connections in the soil food web at higher elevations. Nematode faunal profile showed low nutrient and low disturbance in the region. Carbon footprint of the whole nematode assemblage declined along the elevation. Overall substantial differences in the nematode composition, abundance, trophic structure, and contribution to belowground carbon cycling were observed with change in elevation. These findings could be utilized as useful tool in the long-term monitoring and to understand the region's soil health.

## Introduction

Soil nematodes represent an essential component of the soil faunal community even in the cold ecosystem and can withstand extreme climatic conditions^1–3^. They play crucial role in important soil ecological processes and influences multiple ecosystem functions^4–6^, and are considered potential bio monitoring instrument for ecosystem health^7,8^. Identifying mechanisms for ecosystem health monitoring is very crucial in fragile ecosystems like Himalayan landscape which are under constant threat of climate change. Although in the Indian Himalayan region there are numerous studies on taxonomy, diversity, pest interaction and plant association of nematodes in agroecosystems^9–12^, but only a few studies focused on highlighting the relationship of soil nematodes fauna with elevation gradient in the forest ecosystem of North-Western Himalaya^13,14^.

In the Gangotri landscape, very few taxonomic studies on soil nematode fauna from lower elevation have been reported. However, no attempt has yet been made to study the community structure of nematodes which provide information on the ecological conditions of soils. Therefore, characterization of soil nematode community of such high-altitude wilderness can provide a glimpse of current micro-environmental conditions which may be utilized in the future climate and habitat change studies. It may also elucidate the details of the decomposition pathways therein for long-term monitoring and to understand the region's soil health.

Previous studies have highlighted the elevation pattern of nematode abundance and diversity across temperate forest ecosystems^14,15^, cold desert ecosystems^13,16^, alpine grasslands^17^and sub-arctic vegetations^18^. To the best of our knowledge, no research so far has been carried out to investigate the elevation pattern of soil nematode diversity across high altitude alpine forest ecosystems. So we tried to gain insights about the elevation pattern of soil nematodes along high altitude (3000–5000 m) alpine forest ecosystem.

Here, we specifically aim to answer the following questions.

What is the response of soil nematodes to the increasing elevation? How does the different nematode trophic groups (Bacterivores, Fungivores, Plant parasites, Predators, Omnivores) respond to elevation? Does nematode diversity, abundance and derived functional indices (MI, PPI, CI, SI and EI) which are proxies of their function change with increasing elevation?

## Results

### Soil abiotic characteristics and climatic variables

**Figure 1:**
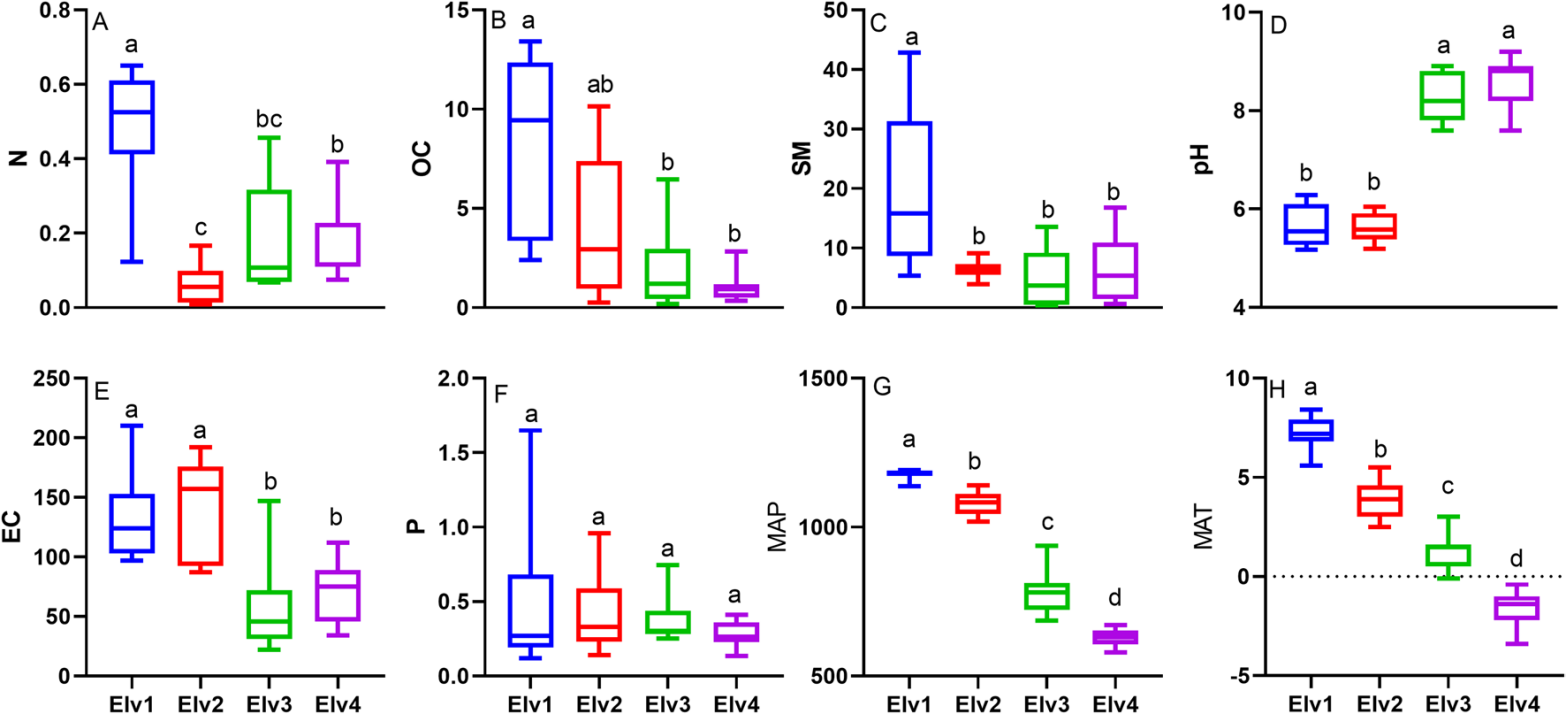
Soil parameter across elevation. Elv—Elevation class; (**A**) N—Nitrogen content (%); (**B**) OC—soil organic carbon (%); (**C**) SM—soil moisture (%); (**D**) Soil pH; (**E**) EC—electric conductivity (µS cm^−1^) and (**F**) P—phosphorus content (%) across elevation classes. Different small case letters across a panel denote significant differences.

Soil nitrogen content, soil moisture and electric conductivity was significantly higher in the lower elevation Elv1 and lowest in highest alpine region of Elv3 and Elv4 (Fig. 1). Additionally, organic carbon was lower in high altitude alpine than lower elevation class Elv1. The pH varied from acidic to alkaline along the elevation gradient. Soil samples from Elv1 and Elv2were found to be acidic but Elv3 and Elv4 with alpine scrub soils were alkaline. There was no significant difference in phosphorous content. MAP and MAT significantly decreased towards high elevation.

### Soil nematode community across elevation

**Figure 2:**
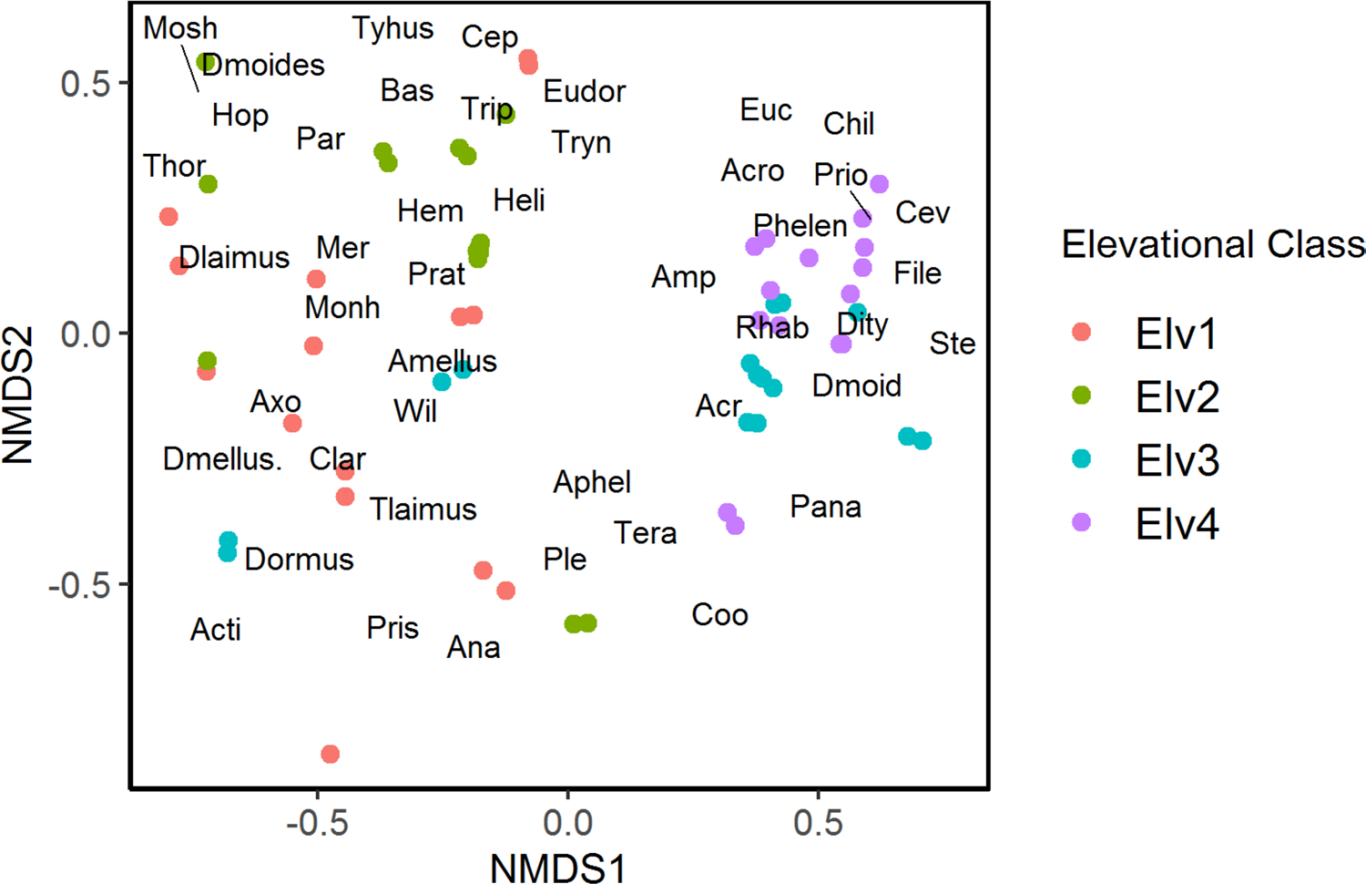
Bray–Curtis dissimilarity (NMDS ordination) of nematode communities based on abundances of nematode genera. Each point displays the community found in an individual sample (n = 15 samples per elevation class × 4 classes = 60). Points that are close proximity have more similar communities than points that are distant from each other. Colors show Elevation class (Where Elv1: 3000–3500 m; Elv2: 3501–4000 m; Elv3: 4001–4500 m; Elv4: 4501–5000 m). Names of nematode genera are overlaid.

**Figure 3:**
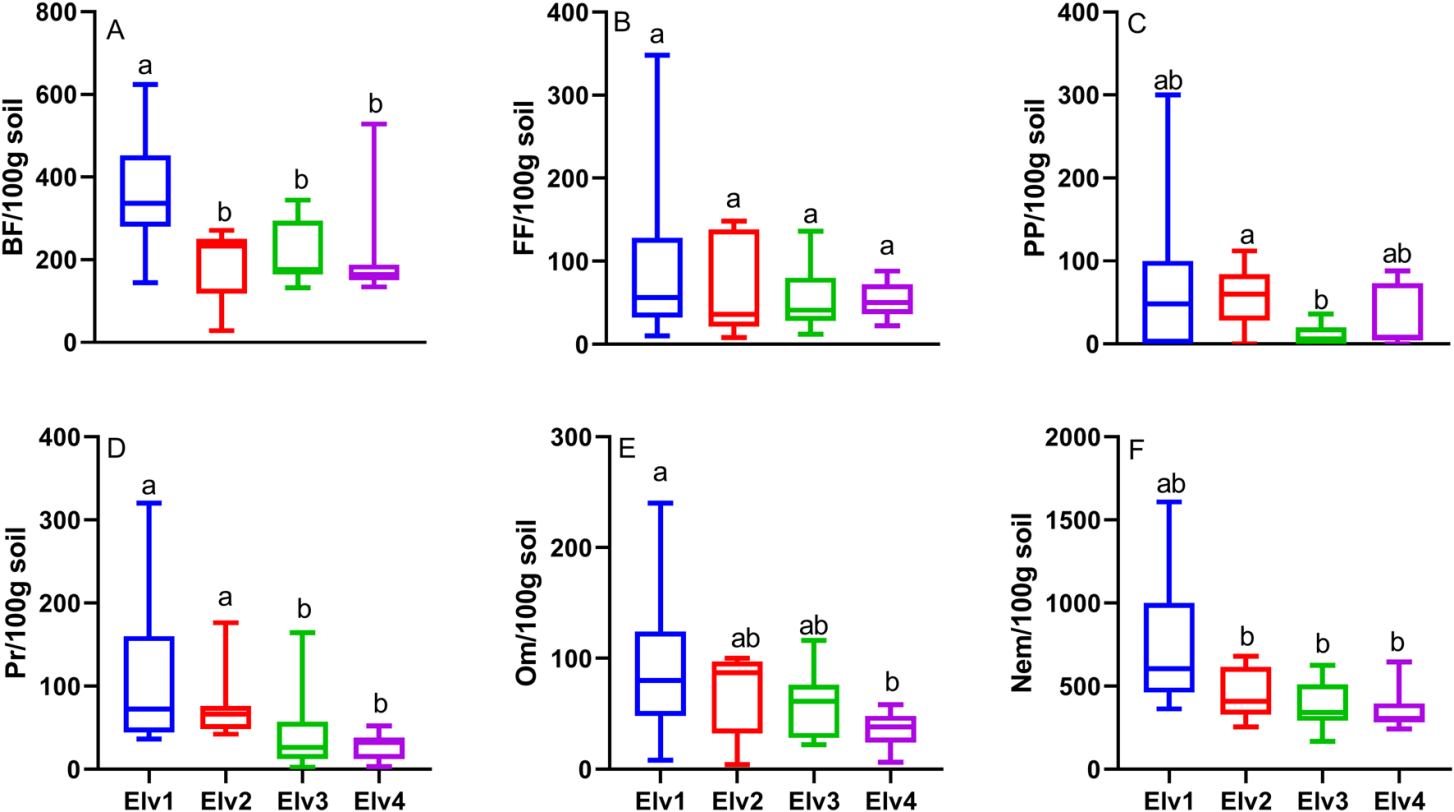
Nematode Abundance of various feeding (trophic) groups across elevation. (**A**) BF (Bacterial Feeders); (**B**) FF (Fungal Feeders); (**C**) He (Herbivores); (**D**) Om (Omnivores); (**E**) Pr (Predators); (**F**) Nem (Total Nematode Abundance). Different small case letters across a panel denote significant differences.

From 60 samples, 28,723 nematodes were assigned to 58 genera belonging to five trophic groups viz. bacterivores-23, fungivores-11, plant parasites-7, omnivores-7 and predators-10 (Table 1). Dissimilarity test based on the Bray–Curtis distance showed that soil inhabiting nematode community structure and taxonomic composition along elevation gradient were significantly different (PERMANOVA, *p* value = 0.001, Fig. 2). Only those genera were plotted that contributed the most to the Bray–Curtis measures between the elevation classes. They are not necessarily different, however to test the significance we compared nematode abundance of these genera across different elevation class with the Kruskal–Wallis test (Table 1). Out of 58 nematode genera, 37genera significantly differed (*p* < 0.05) among various elevation classes (Table 1). 53 genera were recorded from Elv1 (3000–3500 m), 43 from alpine range Elv2 (3501–4000 m) and 39 and 30 genera were recorded from alpine elevation range Elv3 (4001–4500 m) and Elv4 (4501–5000 m) respectively. The mean abundance of soil nematodes varied from 352.6 to 729.3per 100 g soil (Fig. 3).

**Table 1:** Relative abundance of nematode genera in different elevation classes (mean ± standard error).

Genera	Functional guild	Elv1	Elv2	Elv3	Elv4
**Bacteriovores**					
*Acrobeles*	BF_2_	6.79 ± 1.55	5.19 ± 1.07	7.31 ± 1.37	5.22 ± 0.78
*Acrobeloides*	BF_2_	1.92 ± 0.56^a^	5.93 ± 1.54^ab^	11 ± 1.75^b^	6.32 ± 1.2^ab^
*Amphidelus*	BF_2_	0.00 ± 0	0.00 ± 0	0.45 ± 0.28	0.34 ± 0.17
*Anaplectus*	BF_2_	0.57 ± 0.22^a^	0.15 ± 0.1^ab^	0.00 ± 0^b^	0.00 ± 0^b^
*Cephalobus*	BF_2_	1.07 ± 0.49^ab^	4.38 ± 1.02^a^	0.15 ± 0.1^b^	0.41 ± 0.14^b^
*Cervidellus*	BF_2_	1 ± 0.69^a^	1.6 ± 0.61^ab^	6.12 ± 1.47^bc^	12.29 ± 2.51^c^
*Chiloplacus*	BF_2_	2.12 ± 0.67	4.48 ± 0.67	4.01 ± 0.93	7.34 ± 1.08
*Cryptonchus*	BF_2_	0.06 ± 0.04	0.00 ± 0	0.00 ± 0	0.00 ± 0
*Eucephalobus*	BF_2_	0.59 ± 0.34	0.39 ± 0.21	1.33 ± 0.41	1.65 ± 0.4
*Mesorhabditis*	BF_1_	0.53 ± 0.22	0.15 ± 0.1	0.51 ± 0.24	0.00 ± 0
*Monhystera*	BF_2_	0.26 ± 0.12	0.43 ± 0.21	0.24 ± 0.16	0.00 ± 0
*Nothacrobeles*	BF_2_	0.55 ± 0.19	0.56 ± 0.23	0.66 ± 0.36	0.84 ± 0.57
*Panagrolaimus*	BF_1_	0.37 ± 0.14^ab^	0.00 ± 0^a^	2.23 ± 0.47^b^	1.7 ± 0.47^b^
*Protorhabditis*	BF_1_	0.06 ± 0.04	0.00 ± 0	0.00 ± 0	0.00 ± 0
*Plectus*	BF_2_	11.75 ± 2.25^a^	5.73 ± 2.18^b^	6.043 ± 0.40^ab^	5.43 ± 0.52^ab^
*Prismatolaimus*	BF_3_	11.35 ± 2.98^a^	3.01 ± 1.16^ab^	7.13 ± 2.91^ab^	1.96 ± 0.55^b^
*Pseudacrobeles*	BF_2_	0.98 ± 0.22	0.27 ± 0.19	1.71 ± 0.57	1.46 ± 0.61
*Rhabdolaimus*	BF_3_	0.5 ± 0.16	1.03 ± 0.51	2.85 ± 1.12	3.22 ± 0.82
*Rogerus*	BF_2_	0.56 ± 0.41	0.24 ± 0.13	0.00 ± 0	0.00 ± 0
*Stegelletina*	BF_2_	1.01 ± 0.45^a^	1.95 ± 0.42^ab^	3.29 ± 0.86^ab^	4.73 ± 0.7^b^
*Teratocephalus*	BF_3_	2.17 ± 0.69	1.09 ± 0.54	1.59 ± 0.54	3.13 ± 0.4
*Tylocephalus*	BF_2_	0.09 ± 0.07	0.00 ± 0	0.00 ± 0	0.00 ± 0
*Wilsonema*	BF_2_	7.94 ± 2.1^a^	5.52 ± 1.15^a^	1.7 ± 0.56^b^	1.24 ± 0.45^b^
**Fungivores**					
*Aphelenchoides*	FF_2_	2.85 ± 0.75	3.11 ± 0.82	5.94 ± 1.37	3.73 ± 0.62
*Aphelenchus*	FF_2_	0.74 ± 0.3	3.71 ± 1.2	4.31 ± 1.25	0.77 ± 0.35
*Axonchium*	FF_5_	0.69 ± 0.16^a^	0.27 ± 0.19^b^	0.00 ± 0^b^	0.00 ± 0^b^
*Basiria*	PP_2_	0.84 ± 0.34^a^	0.86 ± 0.29^a^	0.00 ± 0^b^	0.00 ± 0^b^
*Coslenchus*	PP_2_	1.45 ± 0.7^a^	0.00 ± 0^b^	0.00 ± 0^b^	2.78 ± 0.86^a^
*Ditylenchus*	FF_2_	0.00 ± 0^a^	0.00 ± 0^ab^	1.43 ± 0.61^bc^	2.3 ± 0.56^c^
*Dorylaimellus*	FF_4_	1.23 ± 0.29^a^	1.53 ± 0.43^a^	0.00 ± 0^b^	0.00 ± 0^b^
*Filenchus*	PP_2_	0.00 ± 0^a^	0.00 ± 0^a^	0.92 ± 0.41^a^	3.21 ± 0.64^b^
*Paraphelenchus*	FF_2_	0.00 ± 0^a^	0.00 ± 0^ab^	1.8 ± 0.77^b^	3.13 ± 0.66^c^
*Tylencholaimus*	FF_4_	0.66 ± 0.17^a^	0.62 ± 0.16^ab^	0.37 ± 0.25^bc^	0.00 ± 0^c^
*Tylenchus*	FF_2_	2.42 ± 0.9^a^	2.35 ± 0.5^a^	0.00 ± 0^b^	0.00 ± 0^b^
**Plant parasites**					
*Helicotylenchus*	PP_3_	1.26 ± 0.56^ab^	2.23 ± 0.44^a^	0.07 ± 0.05^b^	3.41 ± 1.6^ab^
*Hemicycliophora*	PP_3_	1.25 ± 0.68^a^	3.39 ± 0.56^b^	0.56 ± 0.28^a^	1.25 ± 0.86^a^
*Hoplolaimus*	PP_3_	1.26 ± 0.38^a^	2.7 ± 0.44^a^	0.51 ± 0.35^b^	0.00 ± 0^b^
*Merlinius*	PP_3_	0.84 ± 0.43^a^	0.00 ± 0^b^	0.00 ± 0^b^	0.00 ± 0^b^
*Paratylenchus*	PP_3_	0.89 ± 0.4^a^	0.51 ± 0.35^b^	0.00 ± 0^c^	0.00 ± 0^c^
*Pratylenchus*	PP_3_	0.74 ± 0.31^a^	0.00 ± 0^b^	0.00 ± 0^b^	0.00 ± 0^b^
*Tylenchorhynchus*	PP_3_	1.03 ± 0.35	2.7 ± 0.59	1.39 ± 0.42	3.07 ± 0.63
***Omnivores***					
*Dorylaimoides*	Om_4_	1.48 ± 0.46^a^	5.57 ± 0.75^b^	0.24 ± 0.17^c^	0.00 ± 0^c^
*Dorylaimus*	Om_4_	1.82 ± 0.29^a^	0.23 ± 0.23^b^	0.00 ± 0^b^	0.00 ± 0^b^
*Eudorylaimus*	Om_4_	5.64 ± 0.93	6.75 ± 1.19	5.06 ± 0.83	5.57 ± 0.92
*Mesodorylaimus*	Om_4_	0.00 ± 0^a^	0.00 ± 0^a^	3.71 ± 1.39^b^	0.00 ± 0a
*Moshajia*	Om_4_	2.3 ± 0.46^a^	3.23 ± 0.47^a^	0.57 ± 0.26^b^	0.00 ± 0^b^
*Oriverutus*	Om_4_	0.38 ± 0.17^a^	0.00 ± 0^b^	0.00 ± 0^b^	0.00 ± 0^b^
*Thornenema*	Om_5_	1.16 ± 0.24^a^	1.31 ± 0.28^ab^	0.57 ± 0.26^bc^	0.00 ± 0^c^
**Predators**					
*Actinolaimus*	Pr_5_	1.76 ± 0.23^a^	1.13 ± 0.32^b^	0.74 ± 0.36^bc^	0.00 ± 0^c^
*Aporcelaimellus*	Pr_5_	4.87 ± 0.41^a^	4.45 ± 0.47^ab^	3.7 ± 0.59^ab^	1.52 ± 0.62^b^
*Campydora*	Pr_4_	0.28 ± 0.12^a^	0.00 ± 0^b^	0.00 ± 0^b^	0.00 ± 0^b^
*Clarkus*	Pr_4_	0.57 ± 0.22^a^	0.19 ± 0.19^b^	0.00 ± 0^b^	0.00 ± 0^b^
*Coomansus*	Pr_4_	0.14 ± 0.08	0.85 ± 0.54	1.39 ± 0.31	0.68 ± 0.36
*Discolaimus*	Pr_5_	3.06 ± 0.6^a^	3.04 ± 1.26^b^	1.22 ± 0.83^c^	0.00 ± 0^c^
*Discolaimoides*	Pr_5_	1.21 ± 0.38	1.76 ± 0.4	5.42 ± 0.97	5.06 ± 0.86
*Mylonchulus*	Pr_4_	0.17 ± 0.12	0.00 ± 0	0.00 ± 0	0.00 ± 0
*Prionchulus*	Pr_4_	0.21 ± 0.12^a^	0.24 ± 0.13^a^	1.62 ± 0.53^a^	6.24 ± 1.2^b^
*Tripyla*	Pr_3_	0.65 ± 0.27^a^	0.6 ± 0.15^a^	0.00 ± 0^b^	0.00 ± 0^b^

Means (%) followed by different superscript letters are significantly different by the post hoc-test (*p* < 0.05). Means followed by different superscript letters reflects significant differences. Bf: bacterial feeders, Ff: fungal feeders Hr: herbivores, Om: omnivores, Pr: predators and subscript 1–5 represent C–P scale.

There were differences in soil nematode composition among various elevation classes with a significant difference (*p* < 0.05) in the relative abundance of bacterivores (*Acrobeloides*,* Anaplectus*,* Cephalobus*,* Cervidellus*,* Stegelletina*,* Wilsonema*,* Prismatolaimus*,* Plectus* and *Panagrolaimus*); omnivores (*Dorylaimoides*,* Dorylaimus*,* Mesodorylaimus*,* Moshajia* and *Thornenema*) and predators (*Actinolaimus*,* Aporcelaimellus*,* Campydora*,* Clarkus*,* Discolaimus*,* Prionchulus* and* Tripyla*) some Plant parasites and fungivores (Table 1.). Bacterivores (*Anaplectus*,* Rogerus*); fungivores (*Dorylaimellus*,* Axonchium*, and predator (*Tripyla*) were absent in the alpine scrub region of higher elevational range whereas *Protorhabditis*,* Campydora*,* Mylonchulus* and *Oriverutus* were only present in lower elevation class (Elv1).

### Trophic group and c–p group association across elevation

**Figure 4:**
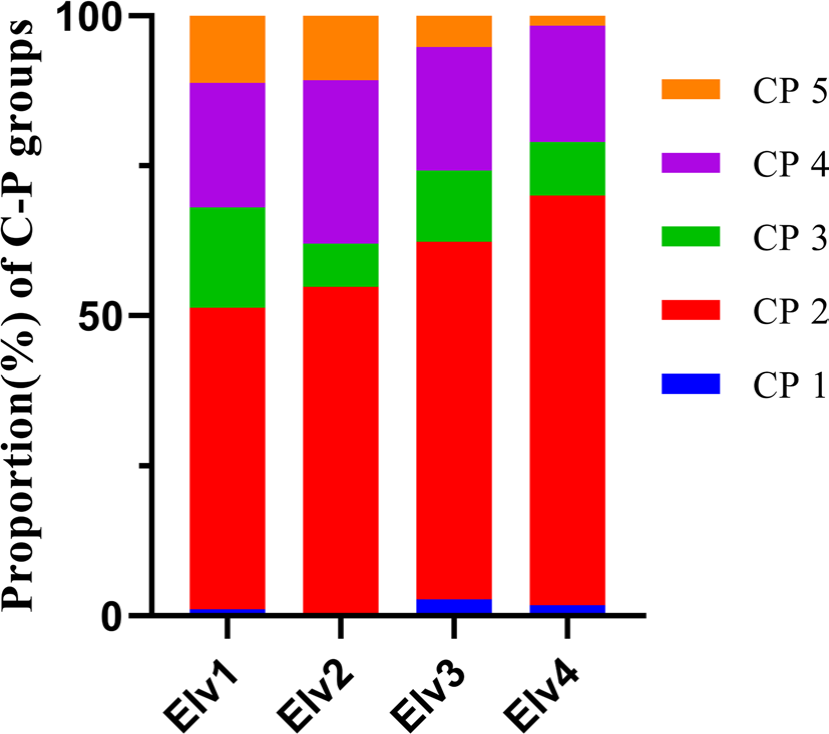
Relative abundance (%) of nematode C–P groups at four different elevation classes.

Trophic groups differed significantly among various elevation classes (*p* < 0.05) except fungivores (Fig. 3). Bacterivores was the most diverse and abundant trophic group across elevation classes, whereas, fungivores were least abundant in highest elevation classes Elv4 (51.73 ± 5.41). Plant parasites were least abundant in Elv3 (10.07 ± 3.11) and were observed in larger numbers in lower elevation classes with subalpine vegetation. Abundance of bacterivores, plant parasites, omnivores and predators were significantly higher in Elv1 among various elevation classes. Nematodes found were also classified into five c–p groups (1–5) according to their colonizer-persister life strategy (Fig. 4). Throughout the elevation classes we found CP 2 nematodes contributing major proportion. However, we found an increasing trend of CP 2 with increasing elevation class and decreasing pattern of CP 5 with increasing elevation class (Fig. 4).

### Nematode community indices across elevation

**Figure 5:**
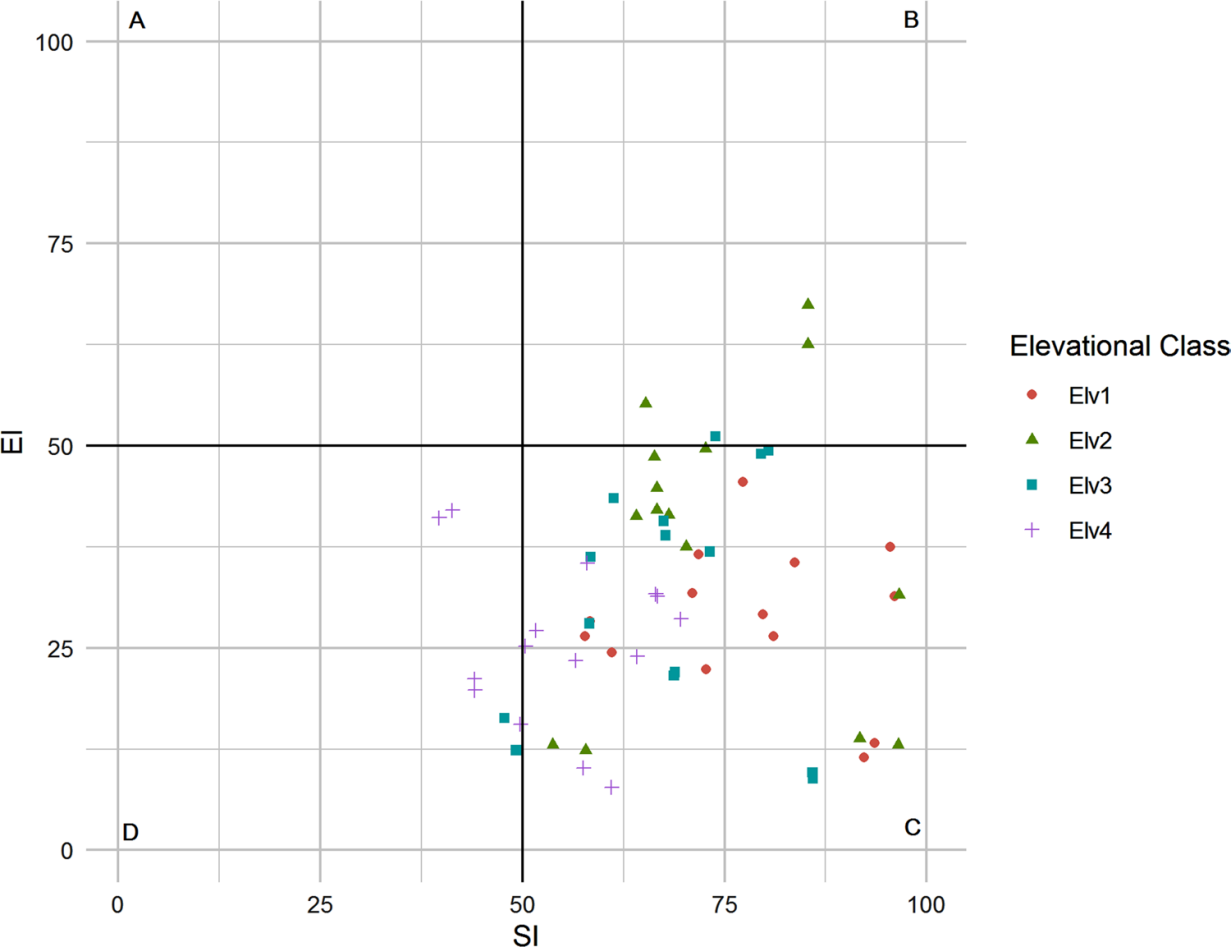
Food web diagnostic across Elevation classes. Elv1: 3000–3500 m; Elv2: 3501–4000 m; Elv3: 4001–4500 m; Elv4: 4501–5000 m.

Maturity indices, significantly varied with elevation with higher values found at Elv1 lower than those found at Elv4 (Table 2). PPI remained unaffected by change in elevation. EI values was significantly higher at elevation Elv3 and Elv4 as compared to Elv1 and Elv2, on the other hand opposite trend was found for SI. CI of soil nematodes were different but not statistically significant among elevation classes. The mean Shannon index (H’) varied among elevation classes with highest value in Elv2 and lowest in Elv3, but the difference was not significant. Evenness index (J′) significantly differed. Nematode Generic Richness (GR) of the overall nematode community was higher at Elv1followed by Elv2, Elv3, Elv4 along the gradient and differences were statistically significant (*p* < 0.05). Nematode faunal profile based on EI-SI, an indicator of ecosystem functioning, for all the elevation classes falls under quadrant C i.e. low nutrient and low disturbance except some of the points of Elv 4 falls under quadrant D (low nutrient and high disturbance) and Elv2 in quadrant 2 (High nutrient, low disturbance) (Fig. 5). Nematode composite structure and various trophic metabolic footprints (Ba, Pp, Pr and Om) significantly varied with elevation, with higher values found at lower elevations (Table 3). Whereas, enrichment and fungivore metabolic footprint remained unaffected by elevation (Table 3).

**Table 2:** Soil nematode community indices across elevation classes.

	Elv1	Elv2	Elv3	Elv4
MI	2.9 5 ± 0.38^a^	2.97 ± 0.45^a^	2.66 ± 0.26^b^	2.51 ± 0.16^b^
ΣMI	2.91 ± 0.32^a^	2.92 ± 0.35^a^	2.67 ± 0.25^b^	2.55 ± 0.15^b^
MI25	2.97 ± 0.37^a^	2.97 ± 0.47^a^	2.71 ± 0.23^a^	2.53 ± 0.17^b^
PPI	2.61 ± 0.32	2.82 ± 0.11	3.08 ± 0.02	3.07 ± 0.08
EI	18.37 ± 13.56^b^	15.22 ± 10.71^b^	28.88 ± 12.03^a^	26.37 ± 7.54^a^
SI	77.16 ± 12.11^a^	75.77 ± 11.25^a^	68.74 ± 9.92^b^	59.85 ± 10.21^b^
CI	64.87 ± 16.72	92.36 ± 17.82	61.27 ± 23.22	73.44 ± 29.62
H′	2.78 ± 0.11	2.84 ± 0.06	2.66 ± 0.07	2.71 ± 0.04
λ	0.9 ± 0.01	0.92 ± 0.01	0.91 ± 0.01	0.92 ± 0.01
J′	0.93 ± 0.01^a^	0.91 ± 0.01^b^	0.9 ± 0.02^ab^	0.91 ± 0.01^b^
Generic richness	27.8 ± 2.59^a^	23.13 ± 0.9^ab^	19.8 ± 0.91^ab^	19.47 ± 0.7^b^

Means followed by different superscript letters reflects significant differences by the post hoc-test (*p* < 0.05).

**Table 3:** Soil nematode metabolic footprints across elevation classes.

	Elv1	Elv2	Elv3	Elv4
Composite footprint	509.63 ± 391.34^a^	222.24 ± 11.56^b^	176.13 ± 143.62^b^	113.24 ± 31.60^b^
Enrichment footprint	8.12 ± 8.45^a^	6.57 ± 7.43^a^	11.86 ± 6.52^a^	9.12 ± 3.14^a^
Structure footprint	439.95 ± 384.64^a^	175.3 ± 122.26^b^	138.5 ± 142.11^b^	74.73 ± 27.21^b^
Plant parasite footprint	16.11 ± 21.03^a^	14.83 ± 8.67^a^	2.01 ± 3.23^b^	4.03 ± 4.95^b^
Fungivore footprint	18.86 ± 15.68^a^	13.44 ± 9.18^a^	20.74 ± 6.32^a^	17.04 ± 7.19^a^
Bacterivore footprint	81.3 ± 32.71^a^	32.71 ± 14.57^b^	41.34 ± 14.83^b^	33.51 ± 16.88^b^
Predator footprint	272.96 ± 291.2^a^	128.83 ± 104.19^b^	91.5 ± 120.98^b^	45.07 ± 23.67^c^
Omnivore footprint	121.41 ± 68.32^a^	32.44 ± 29.61^b^	20.54 ± 14.32b	13.59 ± 8.06^b^

Means followed by different superscript letters reflects significant differences by the post hoc-test (*p* < 0.05).

### Association of soil nematode assemblages with Soil physicochemical parameters across elevation

**Figure 6:**
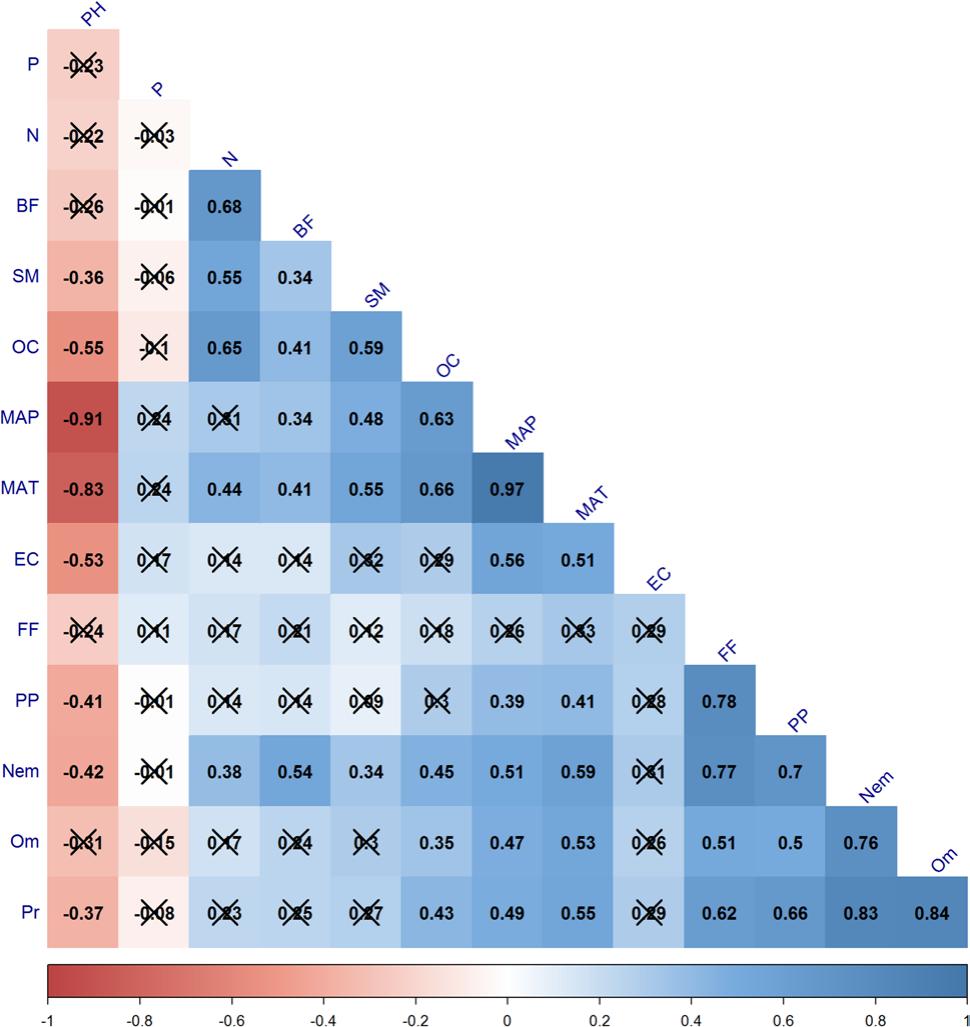
Association of Nematode trophic groups with soil physicochemical parameters. Correlation coefficients (*p* < 0.05) of trophic groups and soil physicochemical parameters. (PH: Soil pH; EC: Electrical Conductivity (µS cm^−1^); OC: Organic Carbon; N: Nitrogen content; P: Phosphorous content; SM: Soil moisture; BF: Bacterivores; FF: Fungivores; Hr: Herbivores; Om—Omnivores; Pr—Predators; Nem—Total Nematode Abundance).

**Figure 7:**
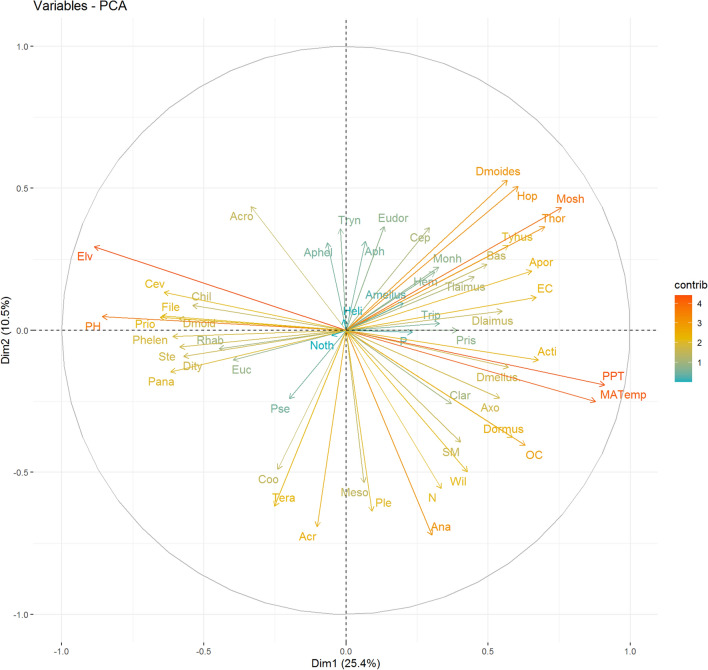
Principal Component Analysis (PCA) of soil Nematode genera trophic groups and soil attributes along elevation. Soil variables elevation trophic group and nematode genera are marked by arrows. Percentage of variance explained was 23.5% and 10.7% for Dim 1 and Dim 2 respectively.

The nematode communities were determined mainly by pH, nitrogen, soil moisture, soil organic carbon contents, and electric conductivity which are mostly related to elevation and vegetation. Soil properties significantly correlated with nematode trophic groups (Fig. 6). Electric conductivity showed significant correlation with fungal feeders. However, various trophic groups showed neutral response with phosphorus content. Abundance of bacterivores showed positive correlation with soil moisture, soil organic carbon and nitrogen content but negatively correlated with soil pH. Omnivores and predators showed positive responses to organic carbon and soil moisture content (Fig. 6). The principal component analysis explains 36.0% of these variations. The first axis explains 25.5% and the second axis explains 10.5% (Fig. 7). The study showed that plots with higher nitrogen, soil moisture and organic carbon are associated with higher faunal diversity of bacterial feeders and genera such as *Anaplectus*,* Plectus*,* Teratocephalus*,* Mesorhabditis*, and *Wilsonema*. The analysis also highlighted that majority of genera showed a negative relation with pH and elevation. However some genera such as *Cervidellus*,* Chiloplacus*,* Filenchus*,* Prionchulus*,* Discolaimoides* and *Paraphelenchus* showed positive correlation with elevation pH and elevation.

## Discussion

Elevation classes signify the variations in plant species assemblages^19^ as a factor of climate, habitat and other environmental variations in nature^20^. In current study, it was observed that soil inhabiting nematode diversity and abundance decreased along the elevation gradient in high altitude region of Gangotri National Park, which is in consistent with findings of previous studies^14,16,21^. It may be due to harsh climatic condition at the higher elevation causing shift in soil abiotic and biotic status which ultimately led to decline in nematode abundance at higher elevation class. In disparity to this, at mid elevation maximum soil nematode diversity pattern have been documented from cold desert^13^. However, some previous studies on soil nematodes along elevation gradient did not find any distinct pattern^3,22^. Moreover, the current investigation showed evidences of significant association of soil nematode trophic group composition and structure with elevation classes in the western Himalayan landscape. It was also observed that among all trophic groups, bacterivores were dominating at all elevation classes although with significant differences at different altitudes. Abundance of plant parasites, omnivores and predators significantly declined along elevation gradient which is in accordance with the previous study in subarctic vegetation^18^. Interestingly previous studies resonate these outcomes specifically in forest areas^23,24^.

The elevation association of nematodes were in agreement with similar studies in the Beskydy Mountains spruce forests, and Changbai Mountain China^25–27^. Yet another study in forest ecosystem linked higher diversity and generic richness at lower elevation forest vegetation soils with its resilient and sustainable ecosystem^28^. So it may be anticipated that the soil ecosystem in lower elevations may be more suitable for sustaining ecosystem balance and soil biota thus resulting in higher generic diversity. Our results depicted that nematode composition in highest elevation class Elv4 is close to Elv3 showing similar nematode composition. It may be concluded that altitude is a significant factor for soil nematode diversity at every elevation class having different local environment and physicochemical properties which affect soil biota specifically, soil nematodes present at various trophic levels in the soil food web.

Diversity of soil nematodes were not solely dependent on altitude but rather on an interactive network of biotic and abiotic compositions of its immediate environment which shifts along the elevation gradient. Ecology of soil nematodes communities are significantly affected by variations in biotic and abiotic factors, external stressors altering the habitat conditions in both forest and agroecosystems^29–33^. External stressors as grazing, forest fire, nutrient supplement (as nitrates phosphates) and alterations in plant types affects the abundance and community composition of the taxa^34–38^. Moreover, soil parameters are also strongly associated with altitude, vegetation, climate and habitat parameters^39–41^. However, reflecting the current results, several studies stated that elevationis vital aspects for determining nematode community composition^21,42^and the aboveground floral composition varies with elevation which is propounded to affect soil inhabiting nematodes by altering abiotic factors^43–46^. In the present study, soil pH levels were found to affect the tropic structure in the study area. Abundance of Plant parasites, omnivores and predatory nematodes were inclined towards more acidic to neutral soils analogous to findings of Keith et al.^47^Apart from pH, soil nematodes were also driven by nitrogen, soil moisture, soil organic carbon contents and electric conductivity which are seldom related to elevation changes along the gradient. Total nematode abundance and bacterivores were significantly associated with nitrogen, soil organic carbon and soil moisture in the current findings. This result was alike numerous studies where bacterivores were higher in nitrogen-rich soil^48–50^.

The maturity indices of soil nematode communities vary from low to high from disturbed to undisturbed soil ecosystems^51^. The present study showed comparatively high disturbance among the highest elevation class with alpine scrubs than the lower elevation class with sub-alpine forest based on maturity indices. EI and SI provided information on soil food web dynamics and energy flow in the system^52,53^ of the present study. It was found that lower elevation range are comparatively nutrient richer than alpine region along elevation which may be due to the adverse conditions at higher elevation directly and indirectly affecting the abiotic and biotic condition and restricted resource entry in the region. Also structural index values differed among ranges which basically indicates higher tropic group nematodes metabolic activity which has crucial function in soil food web^52,54^. Omnivorous and predatory nematodes are known to have a long-life cycle and therefore are highly sensitive to disturbances^51^. But any change in the system affects high trophic level nematodes viz. the predators and this affects the above and belowground stoichiometry^55^. This sensitivity of high trophic soil inhabiting nematodes provides the potential to be used as tool in long-term monitoring and climate change studies to understand the changing soil ecosystem.

Results showed EI-SI for all elevation classes falls under quadrant D i.e. low nutrient and low disturbance except some of the samples from Elv4 fall in quadrant C indicating low nutrient with higher disturbance which is also confirmed by nematode maturity indices. Previous works stated that high SI value of forest soil is owing to high trophic level linkage and high abundance of higher trophic group viz omnivores and predators in soil biota^56,57^. Similar results on EI–SI are known from alpine meadows of the Tatra National Park Slovak Republic^58^. Composition of various nematode species is responsible for maintaining a structured soil food web^59^. Whereas, the mean values of CI in the present study vary (61.27–92.27) among elevation classes indicating variations in dominant groups (bacterial/fungal) of decomposition pathway. Carbon footprint of soil nematodes is lowest at elevations alpine dry scrub. Similar results of carbon assimilation are also reported from temperate vegetation cover along elevation gradient of Pir-Panjal range in Western Himalayas^14^.

Differential nutrient levels among elevation classes and changes in the associated trophic groups of nematodes were evident in the study area. Also nematode diversity and generic richness were relatively higher at a low elevation class of sub-alpine forest and lowest at high elevation alpine dry scrub. Thus nematode diversity and abundance are inversely correlated with elevation. It is known that lower nutrient levels slow down the microbial growth thus directly decelerates the decomposition^48,50,52,60–64^. However, decomposition rates are not regulated by a single parameter rather a combination of temperature, substrate quality, nutrient levels and anaerobic conditions like oxygen and moisture^62,63^ which are mostly associated with altitude and habitat conditions. Among various elevation classes of Gangotri National Park, there are substantial differences in taxonomic composition, energy flow channels and nematode community structure which provide basis to understand the role of soil nematodes in key soil ecological processes in the region.

## Conclusion

The current study presented commendable association among high altitudes soil inhabiting nematode communities of GNP western Himalaya with elevation, soil physicochemical characteristics and soil nutrient levels. Our study showed high nematode richness and abundance in lower altitudinal ranges than the higher ranges. The strong relationship of soil nematode composition and trophic groups with food web dynamics and metabolic footprint can be used in ecological monitoring of the high-altitude ecosystem of western Himalaya. However, before suggesting potential bio indicators, it is essential to establish the ecology of Himalayan soil inhabiting nematode through further extensive systematic studies across the landscape. However, it can be useful in assessing preliminary ecosystem health of forest in the region.

## Material and methods

### Study area

The current study is based on samples collected from the wide altitudinal (3000–5000 m) gradients of Gangotri National Park (GNP) of Uttarakhand in the Western Himalaya. Gangotri National Park lies between 30° 50′ N, 78° 45′ E and 31° 12′ N, 79° 02′ E in the upper catchment of the River Bhagirathi in the Uttarkashi district (Uttarakhand). It covers an area of 2390 km^2^ and a wide altitudinal gradient of 1200–6000 m.

The high-altitude regions of GNP comprise of two major valleys: Gangotri and Nelang Valleys. Gangotri Valley gives rise to River Bhagirathi and Nelang Valley gives rise to one of its largest tributaries River Jadhganga which confluences at Bhairongathi in the Uttarkashi district. The highest elevations covered with ice and bare rocks alpine steppes at altitudes > 3800 m and relatively drier upper region > 3700 m of Gangotri Valley forms a transition zone between moist and arid regions in addition to a stunted tree line between 3700–3800 m. Vegetation of the sub-alpine region is dominated by Himalayan Birch (*Betula utilis*) and sub-alpine Mixed conifer forests of West Himalayan Fir (*Abies pindrow*), Deodar (*Cedrus deodara*) and Blue Pine (*Pinus wallichiana*) classified by Champion and Seth^65^. Deodar primarily dominates the natural vegetation at lower elevations; Blue Pine and Himalayan Birch from mid elevations to tree-line; and alpine scrubs at moist and dry region of the transition zones (*Artemisia sp*,* Juniperus*). However Alpine Dry Scrubs (*Caragana* sp., *Eurotia* sp.,* Rhamnus* sp.,* Artemisia* sp. and* Lonicera* sp.) dominates the cold desert regions of Nelang (4000–5000 m) characterized by rocky terrains, dry soil, very low temperature and scarce rainfall with scanty and specialized vegetation.

### Sampling protocol

Random samplings were conducted along a stratified altitudinal gradient in the study area. Altogether 60 soil samples along elevation gradient (3000–5000 m) were collected in October 2018. Elevation was categorized equally in 500 m class (4 classes) along the altitudinal gradient (Elv1: 3000–3500 m; Elv2: 3501–4000 m; Elv3: 4001–4500 m; Elv4: 4501–5000 m). Three samples per site and a total of 15 samples from each class were collected (3 samples × 5 sites × 4 elevation classes). Five soil cores were taken which constituted one composite sample of 500 gm. At each sampling site beneath the host plant leaf litter (if present) and upper layer of soil were removed. Soil samples were collected at a depth of 0 to 15 cm using a soil auger with a diameter of 3 cm. These samples were then packed in an airtight polythene bag and transferred to the laboratory for further analysis.

### Soil physiochemical parameters and climatic variables

Soil physiochemical parameters viz. Soil pH, Electrical Conductivity (µS cm^−1^), Organic Carbon, N content, NaHCO_3_ extractable P and soil moisture were estimated using standard methods using 350 gm soil sample. Soil pH was determined using a pH meter with glass electrodes according to Singh et al.^66^, Electrical Conductivity (µS cm^−1^) was assessed following Jackson method^67^, whereas, soil organic carbon was estimated by Walkley and Black^68^ method. Total N content was estimated by alkaline potassium permanganate according to Kjeldahl^69^, and NaHCO_3_ extractable P (1954) was estimated using spectrophotometer following Olsen et al.^70^. Lastly, soil moisture content was estimated using standard gravimetric method (replicates were weighed and oven-dried for a day until thoroughly dried and then reweighed again). Temperature and precipitation (mean annual) variables were extracted at a spatial resolution of 30 arc-seconds from WorldClim version 2.1 climate data^71^.

### Nematode extraction and identification

The nematodes were extracted from 100 gm soil samples following Cobb’s sieving and decantation^72^ and modified Baermann’s funnel techniques. The extracted nematodes were fixed in hot triethanolamine-glycerol fixative. Post fixation individual soil nematodes samples placed in petridish were counted under a dissecting microscope. Nematode abundance was expressed as the total number of nematodes per 100 gm of soil sample. To compute the composition of the soil inhabiting nematode community, at least 100 individuals of soil nematodes in each sample were randomly selected after counting^21^. If the total number of nematodes in a sample was less than 100, then all the nematodes were identified upto generic level under microscope (Olympus BX51 DIC Microscope) and were assigned to respective trophic groups^73^. However, the members of the family Tylenchidae owing to their delicate stylet were assigned to fungal feeders according to several authors^14,74^. Nematode genera were also allotted to colonizer–persister (cp) groups^75^.

### Data analysis

Maturity indices were calculated based on a c–p scale assigned to different genera of nematode communities ranging from r-strategist colonizers to K-strategist persisters (cp1–cp5). Maturity Index (MI) and Plant Parasite Index (PPI) were calculated following Bongers^74^. MI-free-living nematodes with cp1-5 excluding plant parasitic nematodes^75,76^, MI25-free living nematodes with cp2-5 excluding nematode with cp1 and plant parasitic nematode; ΣMI25-free-living nematodes and plant parasitic nematodes including nematode with cp-1^77,78^. Enrichment index (EI) = (e/e + b) × 100, Structure index (SI) = (s/s + b) × 100 and Channel Index (CI) = (0.8Fu2/{3.2Ba1 + 0.8Fu2} ×  100) were calculated to provide information on soil food web dynamics and stability^53^. Structure Index is an excellent means to understand whether a system is mature or structured where high value represents structured and low value represents a disturbed ecosystem. Channel index provides data on contribution of bacterial and fungal feeders in the decomposition pathway. The b component is calculated as Σk_b_n_b_ where k_b_ is the weighting assigned to guilds indicating basal characteristics of the soil food web (0.8[Ba2 + Fu2]) and n_b_ is the nematode abundance in the guilds. Similarly, enrichment and structure components were calculated using guilds indicating enrichment ([3.2Ba1] + [0.8Fu2]) and structure (Ba3–Ba5, Fu3–Fu5, Ca2–Ca5) respectively^52^, where Ba1-5 represent bacterivores of C–P group 1–5, Fu2-5 represent fungivores of C–P group 2–5 and Ca2-5 represent predators of C–P group 1–5 All the nematode functional indices were calculated using the online program NINJA^79^.

Shannon–Wiener diversity index (H'); evennessindex (J′); and Genera richness (GR) were used for quantification of diversity. The metabolic footprint (carbon assimilation) which are proxies of magnitude of ecosystem services and functions provided by nematodes to the soil food web were calculated with the help of equations provided by Andrassy^80^: *W* = (*D*^2^ × *L*)/(1.6 × 10^6^) and Ferris^81^:*F* = Σ(*N*_*t*_ (0.1(*W*_*t*_/*m*_*t*_) + 0.273 (*W*_*t*_0.75)) using the NINJA online program^77^.

As our data was not normal even after transformation therefore, Kruskal–Wallis Analysis of Variance (ANOVA) was used to detect significant differences in nematode abundance, diversity, nematode specific indices and soil properties among different elevational classes. Multiple comparisons among elevational classes were performed by using the Dunn test. Spearman's rank correlation coefficient and its significance at *p* < 0.05 level among various soil physicochemical parameters, climatic variables and nematode trophic groups were calculated. To establish a relation between physicochemical properties of soil, climatic variables, trophic groups and nematode genera Principal component analysis (PCA) was used. Non-Metric Multidimensional Scaling (NMDS) was performed to investigate the position of soil nematode community in ordination space along elevation gradient. Permutational Multivariate Analysis of Variance (PERMANOVA) was used to test if nematode composition differs along elevation gradients using ‘adonis’ and ‘betadisper’ functions of vegan package (R 3.6)^82^. Pairwise multi-level comparisons were conducted with dissimilarity matrix using ‘pairwise.adonis’ function to assess differences between elevations. All analyses were performed in R (version 3.6) statistical platform (R core team)^83^. Figures 1, 3 and 4 were prepared with the help of software GraphPad Prism8.0.25^84^.

## Abbreviations

MI: Maturity Index
CI: Channel Index
EI: Enrichment Index
SI: Structure Index
